# Superficial myofibroblastoma of the genital tract: a case report of the imaging findings

**DOI:** 10.1259/bjrcr.20180057

**Published:** 2018-08-11

**Authors:** Angela Atinga, Mona El-Bahrawy, Victoria Stewart, Nishat Bharwani

**Affiliations:** 1 Department of Imaging, Imperial College NHS Trust, London, UK; 2 Department of Histology, Imperial College NHS Trust, London, UK; 3 Department of Surgery and Cancer, Imperial College London, London, UK

## Abstract

Superficial angiomyofibroblastomas are mesenchymal tumours that occur in the genital tract and are well described pathologically. This case report reviews the imaging appearances and highlights the MRI findings, which have not been previously described. We describe the occurrence of this lesion in a vaginal cyst, which to the authors’ knowledge, has also not been previously described. The histological findings are also presented here.

## Introduction

Mesenchymal tumours of the female genital tract are well described pathologically but the imaging findings are less well documented. We present a case of an unusual mesenchymal tumour of the female genital tract, highlight the key sonographic and MRI findings and summarise the current diagnostic and management approach.

## Clinical history

A 50-year-old female presented with a history of prolonged menstrual bleeding and right iliac fossa discomfort. Her past medical history of note included endometriosis, a partially septate uterus and two previous lower segment cesarean sections. She had no allergies and denied any relevant drug history such as tamoxifen or hormonal therapy use.

## Imaging findings

**Figure 1.**
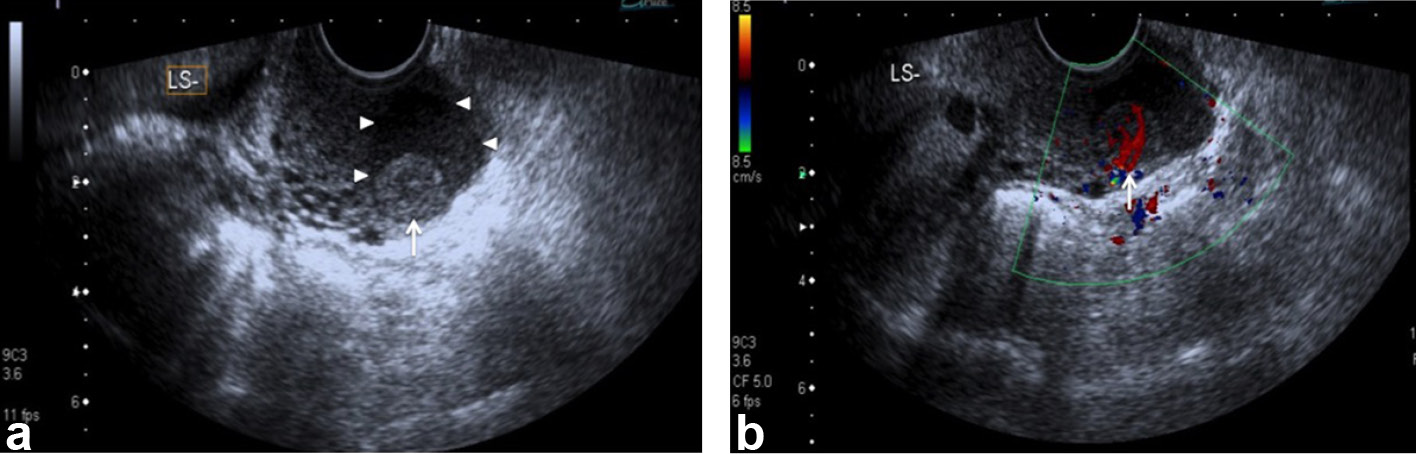
(a and b) Transvaginal B-mode ultrasound demonstrates a 25 mm cystic lesion (arrowheads) in the left lateral vaginal fornix with low-level internal echoes and a 13 mm solid nodule (white arrow), which is hypervascular on Doppler ultrasound. The uterus and ovaries were sonographically normal.

Initial pelvic ultrasound demonstrated normal uterus and ovaries, but detected a 13 mm vascular soft tissue nodule in a 28 × 20 × 25 mm cystic lesion in the left posterior vaginal fornix (Figure 1). The differential diagnosis at this time included a cervical or high vaginal polyp or an endometrioma, although the solid vascular component was unusual for the latter.

**Figure 2.**
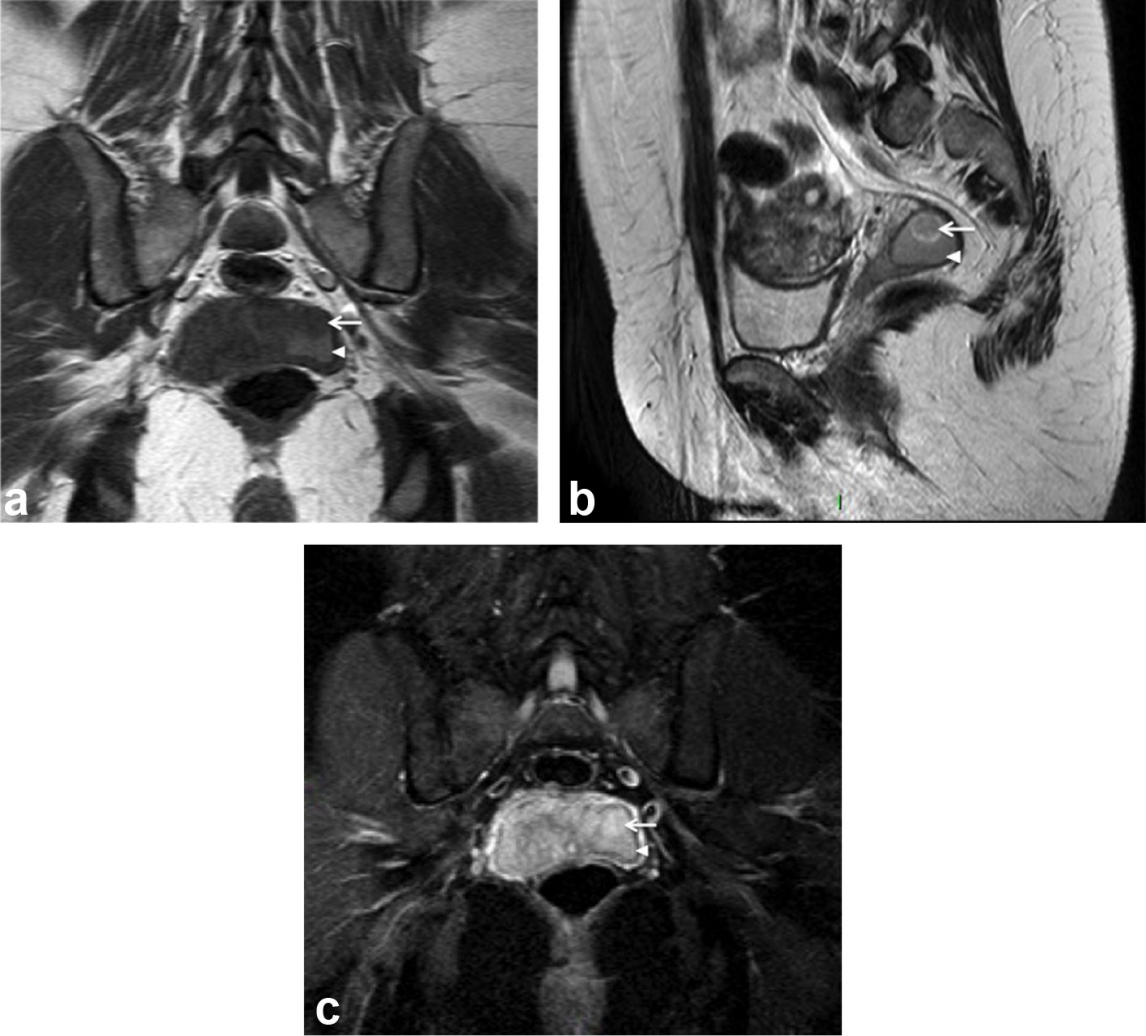
(a–c) Pelvic MRI study shows a well-defined cystic structure (arrowhead) in the left vaginal fornix, which demonstrates mildly hyperintense internal signal on T1W images (a) and high signal on T2W images (b). The internal solid component (white arrow) had a T2 hyperintense rim and was hypointense on T1W images. There was no signal suppression on the STIR images (c) to suggest a fatty component. STIR, Short T1 Inversion Recovery; T1W, T1 weighted; T2W, T2 weighted.

The patient proceeded to have a non-contrast enhanced MR study. This confirmed a well-defined, predominantly cystic, 24 × 31 × 24 mm structure in the left vaginal fornix of mildly hyperintense signal on T2 weighted (T2W) images, intermediate to high signal on T1 weighted (T1W) images with a mild increase in signal intensity on T1W fat-suppressed images. The internal solid component was of intermediate signal with a hyperintense rim on T2W images and low signal on T1W images. There was no signal suppression on the Short T1 Inversion Recovery (STIR) images to suggest a fatty component (Figure 2). The lesion was not thought to be suspicious and, as the patient was asymptomatic, no intervention was undertaken at the time. Two years later, the patient returned with persistent vaginal bleeding and repeat imaging showed the cystic component had increased in size and now measured 38 × 44 × 38 mm, but there was no change in the solid component.

## Surgery and histology

The patient underwent total laparoscopic hysterectomy and bilateral salpingo-oophorectomy. The mixed solid cystic vaginal lesion was excised at the same time.

Gross examination revealed a nodular well-circumscribed lesion measuring 16 × 12 × 14 mm and a small tissue fragment measuring 5 × 6 × 2 mm. Both specimens were submitted to the laboratory labeled as vaginal cyst.

**Figure 3.**
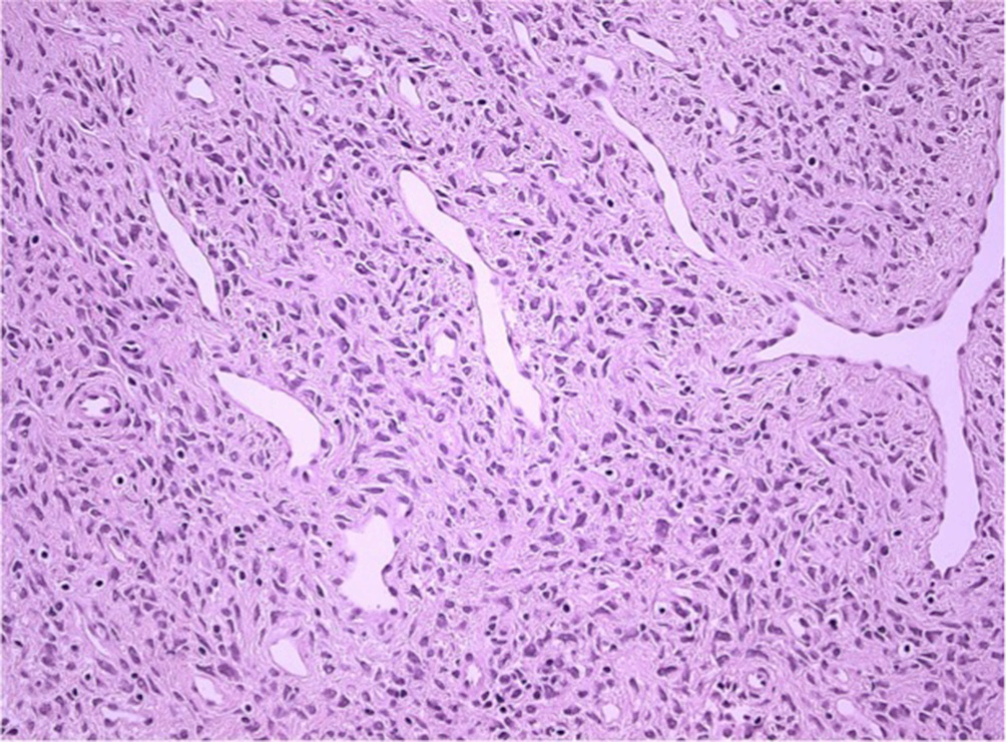
Histological features of superficial angiomyofibroblastoma: The lesion is composed of bland spindle cells with pale eosinophilic cytoplasm set in stroma rich in thin-walled vascular channels (× 200).

Microscopically, the small tissue fragment was a strip of tissue lined by stratified squamous epithelium with little underlying stroma. The nodule was a well-circumscribed lesion comprised of predominantly bland spindle cells with pale eosinophilic cytoplasm and ill-defined cell borders, set in loose focally oedematous stroma containing abundant thin-walled vascular channels (Figure 3). There were no mitoses and no evidence of necrosis, but the tumour extended to the excision margin.

Immunohistochemical analysis showed the tumour cells were positive for ER, PgR, CD34, Desmin, CD99, BCL-2 and negative for H-caldesmon, SMA, c-Kit, S100 protein, MNF116, HMB45 and melan A. Less than 2% of cells were positive for Ki67.

The uterus and fallopian tubes were normal and both ovaries displayed cystic changes, but there was no evidence of malignancy. The accompanying peritoneal washings were also negative.

A histological diagnosis of superficial myofibroblastoma of the lower female genital tract was made.

## Follow up

Sonographic surveillance over a period of 8 years has not demonstrated local recurrence.

## Discussion

Superficial myofibroblastomas of the female genital tract are rare tumours but are well documented histologically. They preferentially occur in the lower female genital tract, with majority of cases in the vagina, cervix or vulva.^1, 2^ The tumours have been described in patients with a wide age range^1^ and there is at least one reported case of the tumour presenting in pregnancy.^3^

The classical histological description is of a tumour with bland spindle cells that are weakly eosinophilic, set in loose stroma with multiple vascular channels, oedematous foci and scattered inflammatory cells including lymphocytes and macrophages.^1, 2,4^ The tumours also have a very characteristic immunohistochemical profile and are positive for oestrogen and progesterone, CD34, vimentin and desmin,^1, 2^ but do not express smooth muscle actin, which distinguishes them from leiomyomas.^5^

Although these tumours do not have any malignant potential,^1^ they can recur locally with one series demonstrating recurrence after a period of 9 years,^6^ therefore long-term follow up is advised.

The aetiology of the tumours remains unclear. Approximately 94% of the reported cases (32 of 35 in 2010) occurred in peri-menopausal or menopausal females, and 32% of patients (11 of 35) had a history of tamoxifen or HRT use.^1, 7^ Ganesan et al postulated that tamoxifen may drive growth through the estrogen receptor or alternatively, patients on tamoxifen therapy are likely to be more vigilant as they are under surveillance for endometrial changes, so increasing the detection rate.^1^

Mesenchymal tumours in other locations have been linked to viral infections but to date, only one study has addressed this possibility in myofibroblastomas of the female genital tract and revealed no association to human papilloma virus, human herpes virus 8 or Epstein Barr virus.^8^

The imaging features have not been previously described in the medical literature. Sonographically, the tumour in our case was a hyperechoic solid mass with intense intralesional vascularity and demonstrated slow interval growth (Figure 1). The soft tissue nodule was isointense to muscle on T1W, intermediate signal intensity with a hyperintense rim on T2W images, and of high signal on the STIR sequences (Figure 2). Interestingly, the tumour in our patient occurred within a thin-walled vaginal cyst, which has not been previously described.

The finding of intralesional vascularity excludes lesions such as Bartholin cysts, rectoceles or urethral diverticula.^7^ It also makes the potential diagnosis of a simple endometriotic cyst unlikely as these have been shown to be purely cystic with diffuse internal echoes and scanty vascularity on ultrasound.^9^ The polypoidal, superficial cervical and vaginal subtypes of endometriosis have a more solid soft tissue component, but their immunohistochemical profile differs from that of the superficial myofibroblastoma.^10^

There is considerable overlap in the imaging and histological features of many of the mesenchymal tumours of the genital tract. The majority of diagnostic strategies are aimed at distinguishing lesions on the benign end of the spectrum from the more aggressive angiomyxoma subtype, which is invasive and warrants a different management strategy. There is a subset of tumours that are relatively site-specific and arise from the superficial tissues of the genital tract.^7, 11^ These include aggressive angiomyxoma, angiomyofibroma, fibroblastoma and cellular angiofibromas, as well as the tumour described in our case, superficial myofibroblastoma. The second subset of tumours that frequently occur in this region but also occur elsewhere includes fibroepithelial stromal polyps, leiomyomas and superficial angiomyxomas.^7^

Angiomyofibroblastoma subtypes are well-defined, homogeneous vascular lesions of medium echogenicity on ultrasound and are homogeneously low intensity solid masses on T2W images.^11^ Cellular angiofibromas, also known as angiomyofibroblastoma-like tumour, present as well-circumscribed tumours of inhomogeneous echotexture but are relatively isoechoic to surrounding subcutaneous fat. On MRI, the tumours are isointense or hypointense to muscle on T1W images, heterogeneous intermediate to high signal on T2W images with scattered low signal areas depending on the fibrous tissue component, and enhance following administration of Gadolinium.^12^ The majority of the more aggressive angiomyxoma subtypes are well-defined, hypoechoic, cystic or multiseptate vascular masses on ultrasound, of high T2 signal on MRI and have a swirled or lamellate appearance.^13, 14^

Ultimately, definitive diagnosis requires surgical excision and careful histological examination. Long-term imaging follow up is recommended, as there has been at least one case of recurrence after a period of 9 years.^6^ Our patient has been free of recurrence for 8 years.

## Conclusion

We have presented a unique case of superficial myofibroblastoma of the lower genital tract and discussed the imaging findings and main differential diagnosis.

## Learning points

1. Superficial myofibroblastomas are mesenchymal tumours that occur preferentially in the female genital tract.
2. The less aggressive spectrum of tumours appear to have a more solid, hyperechoic appearance on ultrasound and characteristic MR features when compared to the more aggressive angiomyxoma subtypes which may be hypoechoic, cystic or septated; however, definitive diagnosis based on imaging alone is not possible and surgical resection is required.
3. These tumours may be locally aggressive and recur locally; therefore, follow-up imaging is advised, although there is no consensus about the optimum length of time at present.
4. The authors declare no conflicts of interest and have obtained written informed consent from all parties involved in this publication.
